# The spatial characteristics of plaid-form-selective mechanisms

**DOI:** 10.1016/j.visres.2010.01.018

**Published:** 2010-04-07

**Authors:** David P. McGovern, Jonathan W. Peirce

**Affiliations:** Nottingham Visual Neuroscience, School of Psychology, University of Nottingham, Nottingham NG7 2RD, United Kingdom

**Keywords:** Adaptation, Contrast, Phase, Mid-level

## Abstract

Rather little is known about the mechanisms that combine the outputs of orientation- and spatial frequency-selective channels. These can be studied by measuring the selective adaptation to compound stimuli over and above that expected from adaptation to the components alone (Peirce & Taylor, 2006). Here we investigated the contrast- and spatial phase-dependency of such mechanisms. A plaid was adapted in one visual hemi-field, while its constituent gratings were simultaneously adapted in the other hemi-field. Plaid-selective adaptation was most apparent with high-contrast probes, whereas adaptation to the component grating stimuli dominated at low contrasts. The mechanisms underlying this plaid-selective adaptation also appear to be insensitive to the spatial phase of the probes relative to the adaptor, whereas we find a clear phase-dependency for suprathreshold contrast adaptation to grating stimuli. These findings suggest that the visual system is equipped with mechanisms that conduct a global analysis of the plaid pattern, which are likely derived from the non-linear outputs of V1 complex cells.

## 1 Introduction

Physiological studies suggest that shape processing involves a hierarchy of processing stages. Neurons located at the beginning of this hierarchy, in primary visual cortex (V1), respond to bar stimuli and sinusoidal gratings (Hubel & Wiesel, 1968) while much higher in the hierarchy, cells in inferotemporal cortex (IT) respond to more complex shapes, such as faces and stars (Gross, Rocha-Miranda, & Bender, 1972; Perrett, Rolls, & Caan, 1982; Tanaka, 1993, 1996). The function of neurons located in mid-level areas V2 and V4 remain less clear (Boynton & Hegde, 2004; Wilkinson et al., 2000). V4 cells have also been found to respond to moderately complex stimuli including polar and hyperbolic gratings (Gallant, Braun, & Van Essen, 1993; Gallant, Connor, Rakshit, Lewis, & Van Essen, 1996) and blobs that vary in their curvature (Pasupathy & Connor, 1999; Pasupathy & Connor, 2002). It appears that V2 cells also respond to contours comprised of line segments, dependent on the angle formed between the components (Ito & Komatsu, 2004; Kobatake & Tanaka, 1994) and to the presence of second-order stimuli such as ‘illusory contours’ (von der Heydt, Peterhans, & Baumgartner, 1984).

Psychophysical adaptation studies have also been used to demonstrate the selectivity of mechanisms found in the visual system. For instance, the effects of neurons responding selectively to stimuli of a particular orientation and spatial frequency (SF) have been shown by adapting subjects to particular stimuli and showing that the effect of this adaptation on detection thresholds is selective (Blakemore & Campbell, 1969; Snowden & Hammett, 1992; Tolhurst & Barfield, 1978). Similar findings show that suprathreshold stimuli have a lower apparent contrast after adaptation to a high-contrast grating of a matching spatial frequency, although the effects on suprathreshold probes may be less selective in the spatial frequency domain (Blakemore, Muncey, & Ridley, 1973; Georgeson, 1985; Snowden & Hammett, 1992, 1996). These shifts in sensitivity occur even after brief exposure to an adapting grating (Georgeson & Georgeson, 1987), although the duration of these aftereffects is much shorter than that induced by prolonged exposure of the adaptor (Magnussen & Greenlee, 1985). Furthermore, there is some evidence to suggest that contrast adaptation may enhance contrast discrimination (Abbonizio, Langley, & Clifford, 2002; Greenlee & Heitger, 1988 but see Määttänen & Koenderink, 1991). These findings taken together suggest that adaptation acts to adjust the shape of the underlying mechanism’s contrast response function, in order to increase its dynamic range.

More recent research has used adaptation as a tool for examining the mechanisms that combine the outputs of V1 neurons. Some of these studies have focused on the mechanisms that encode curvature (Gheorghiu & Kingdom, 2006, 2007; Hancock & Peirce, 2008), while others have investigated those that encode the form of plaid patterns (Georgeson & Meese, 1997; Meese & Georgeson, 1996; Peirce & Taylor, 2006). Although plaids have more typically been used to study the combination of motion signals (Huk & Heeger, 2002; Movshon, Adelson, Gizzi, & Newsome, 1986; Rust, Mante, Simoncelli, & Movshon, 2006) they are also an ideal stimulus with which to study the combination of form signals, being the simplest possible conjunction of two oriented components. Peirce and Taylor (2006) used a method of compound adaptation, whereby participants simultaneously adapted to multiple sinusoidal plaid patterns separated across the two visual hemi-fields. The two fields contained identical grating components (A, B, C, D) rearranged into different plaid patterns (AB, CD and AC, BD). Participants then compared the contrast of a plaid in the region where it had been adapted as an intact pattern (the compound field) with another region where the pattern’s components had been presented to an equal extent, but separately, forming other plaids (the component field). The results showed that the test plaid appeared to have a lower apparent contrast in the compound-adapted field than in the component-adapted field indicative of mechanisms selective to the form of the pattern rather than to the individual gratings forming the plaid (Peirce & Taylor, 2006). These plaid-form-selective mechanisms may (Movshon et al., 1986) or may not (Rust et al., 2006) be a precursor to plaid-motion-selective mechanisms found in area MT/V5.

Although Peirce and Taylor’s results suggest the presence of plaid-form-selective mechanisms, it does not tell us how they are built from V1 receptive fields. In order to understand the nature of these mechanisms more fully, we investigated the effects of probe contrast and spatial phase on the compound adaptation effect, using a variant of the original method (for a demo see Movie 1). In Experiment 1, we simultaneously adapted participants to high-contrast gratings and plaid stimuli (in different visual hemi-fields) and tested them with probes of a range of different contrasts. Our results show that adaptation to the grating stimuli was more apparent with low probe contrasts, whereas selective adaptation to the plaid was greater for high probe contrasts. This difference between the adaptation to grating and plaid stimuli suggests differences between the contrast response functions of the neurons that process these stimuli. Moreover, the unusual trend of these adaptation effects suggest that the visual system employs an efficient coding strategy to encode plaid patterns, attenuating the response of neurons to simple gratings once a compound is detected (e.g. Murray, Kersten, Olshausen, Schrater, and Woods, 2002; Fang, Kersten, and Murray, 2008).

We also examined the effect of phase on compound adaptation, in order to determine the nature of the sub-units that form these plaid mechanisms. To facilitate direct comparison of phase tuning for plaid and grating stimuli, we first examined whether varying the spatial phase of a probe relative to the adaptor had any impact on adaptation to a grating stimulus. Previous studies have shown that adaptation-induced threshold elevations occur independently of phase differences between adaptor and test grating patterns (Jones & Tulunay-Keesey, 1980). However, these experiments were performed at threshold and a different result might be expected in a suprathreshold matching paradigm (e.g. Hol & Treue, 2001) such as the one employed here. Indeed, our results show that observers demonstrate selectivity for spatial phase. Adaptation was most apparent when the test probe was presented at the same spatial phase as the adaptor, while a diminished aftereffect was observed when the phase of the probe was shifted away from the adaptor. This was not the case for plaid-selective adaptation. The spatial phase of the probe relative to the adaptor had no impact on the magnitude of adaptation effect for the plaid pattern, suggesting that the underlying mechanism is phase invariant. Together, these findings suggest that the visual system is equipped with mechanisms that encode the form of the plaid pattern, which are qualitatively different from the oriented filters of V1.

## 2 Methods

The stimuli used in Peirce and Taylor (2006) consisted of four different sinusoidal gratings combined together to make four different plaids. For this set of experiments a simpler stimulus set was used. Participants were adapted to two alternating gratings (contrast = 0.5) on one side of fixation (the *component field*), while adapting to a full-contrast plaid comprising the same components on the other (the *compound field*). In the compound field the plaid alternates with a blank grey screen such that the overall exposure to the components is constant in the two locations. The participant was then presented with a test probe in both hemi-fields at the same time and was required to report which side had a higher apparent contrast.

### 2.1 Participants

Participants consisted of three healthy volunteers (two experienced observers and one naïve participant) with normal or corrected-to-normal vision who gave their consent.

### 2.2 Apparatus

Stimuli were presented on a computer-controlled cathode-ray-tube (CRT) monitor (Vision Master Pro 514, liyama) at a resolution of 1152 × 864 pixels and at a refresh rate of 85 Hz with a mean luminance of 108.3 cd/m^2^. A photo-spectrometer (PR650, Photo Research, Chatsworth, CA, USA) was used to gamma-correct the red, green and blue (RGB) guns independently and the gamma correction was verified psychophysically using a 2nd-order motion-nulling procedure (Ledgeway & Smith, 1994). The monitor was driven by 14-bit digital-to-analogue converters (DACs) (Bits++, Cambridge Research Systems, Cambridge, UK). Stimuli were presented and data collected using the PsychoPy stimulus generation library (Peirce, 2007b). The observer’s head was stabilized in a chin-rest 57 cm from the monitor with the viewable area subtending 40.5° of visual angle.

### 2.3 Stimuli

Plaids were constructed from the linear combination of two luminance-modulated sinusoidal gratings, one oriented vertically and one horizontally, both with spatial frequency (SF) = 0.8 c/°. The component gratings contributed equal contrast to the plaid. All stimuli were presented in a Gaussian envelope with a standard deviation 0.5° (such that the stimulus had a diameter of 5° at the point where it fell below 1% contrast).

### 2.4 Procedure

Participants were adapted to alternating gratings and to the plaid simultaneously at different positions on the retina (centred at 3° either side of the fovea on the horizontal meridian). In the component field, gratings were presented at a contrast of 0.5 alternating between gratings every second. In the compound field, the gratings were summed (giving rise to a full-contrast plaid) and alternated with a blank grey field every second. See Movie 1 for a demo of the effect. Note that the resulting adaptation to the components should be identical in the two fields, given that the adaptors have equal Fourier energies. The observers then compared the contrast of a probe (one of the gratings or the plaid) at the same retinal location that it had itself been adapted (the test probe) with one in the opposite location (the reference probe) and were required to report which side had the higher apparent contrast. The reference probe took a fixed contrast value, while the contrast of the test probe gradually decreased or increased in steps using an adaptive 1-up, 1-down staircase procedure designed to maintain stimulus presentation near the point of subjective equality (PSE). Staircases were randomly interleaved for the three probe types (each grating and the plaid). Step sizes at the beginning of a trial were large (8 dB) and gradually decreased (4, 2, 1 and 0.5 dB) as the participants responded in order to home in on the point of subjective equality.

**Fig. 1:**
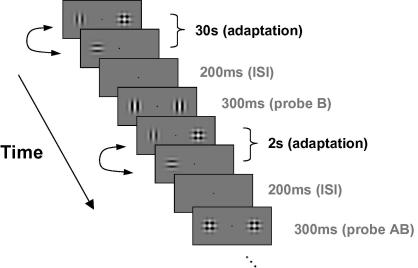
Schematic diagram of procedure. After an initial adaptation period of 30 s, participants were presented with a probe for 300 ms and were required to respond which side had the higher apparent contrast. This was followed by a 2 s “top-up” period before the presentation of another probe.

In all experiments there was an initial adaptation period of 30 s with a 2 s “top-up” period before each trial. A blank inter-stimulus interval (ISI) with a duration of 200 ms followed before the presentation of the probes for a further 300 ms (see Fig. 1 for a full schematic of the procedure). The fixation point was presented at the centre of the display (between the component and compound fields) and was visible for the entire experiment. The participant responded by pressing either the cursor keys on a standard keyboard to indicate which hemi-field had the higher contrast. The response triggered the next top-up period and trial. A single run comprised of 50 trials for each probe type, randomly interleaved. Subjects conducted a total of eight runs per condition (adapting with the compound field in the left and right hemi-fields four times each). On any day of testing participants were only adapted to the plaid in one hemi-field.

Experiment 1 aimed to measure the dependency of the plaid adaptation effect on the contrast of the probe. The spatial phase of the adapting stimulus was jittered (taking a new random value every 200 ms) across time to prevent retinal afterimages. The reference probe took one of five Michelson contrast values 0.15, 0.21, 0.30, 0.42 and 0.60 depending on the trial.

Experiment 2 aimed to measure the phase-dependency of the effect. The adapting stimuli were presented with the same contrast and timing parameters as in Experiment 1, but were not phase-jittered. Instead, stimuli were counterphase modulated at a rate of 5 Hz to prevent retinal afterimages. The spatial phase of the probe relative to the adaptor took one of five phase values ranging from 0° to 90°, depending on the trial, that increased in steps of 22.5°. Since the counter-phasing procedure effectively modulated the stimuli between 0° and 180° any effect of the relative phase of the probes should be symmetric about a phase of 90°. Reference probes had a fixed contrast value of 0.42. All stimuli were comfortably suprathreshold.

### 2.5 Data analysis

**Fig. 2:**
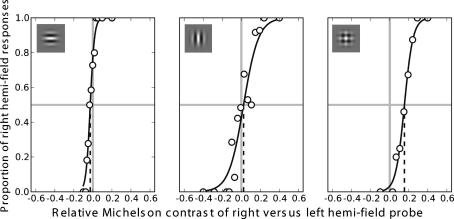
Psychometric function for one observer (SH) for trials that used a reference probe with a fixed Michelson contrast of 0.6. In this block of trials the plaid AB was adapted in the right visual hemi-field while the components were adapted on the left. For the component probe the PSE occurs when the probes are genuinely similar, consistent with the roughly equal component adaptation in both visual hemi-fields. For the plaid probe, however, the PSE occurs when the right-hand probe has a contrast of 0.76 versus 0.6 in the left, indicating that the plaid was more strongly adapted by the compound pattern adaptor. Note that, although for this subject there was a difference in the slopes of the psychometric function between component probes, this was not generally the case.

Data from the 200 trials per condition and side of compound adaptor (4 × 50-trial staircases) were combined in the following manner. For each test-probe contrast presented during the staircase procedures, the outcomes of all trials were averaged resulting in a complete psychometric function (see Fig. 2). A Weibull function was fitted to these averaged data, from which we determined the PSE, representing the additional contrast needed in the test probe for the test and reference probes to appear equal. This PSE is calculated for trials on each side and averaged to remove any effects of response biases with respect to visual hemi-field. This average value we take to represent the degree of selective adaptation to the compound (i.e. above that which can be explained by adaptation to the component gratings). In a similar manner, selective adaptation to the grating probes is the average additional adaptation found in the component field that cannot be explained by adaptation to the compound.

We generated 5000 bootstrap resamples of the data (each with 200 trials as in the original dataset for each condition and side). A Weibull function was fitted to the bootstrapped resamples as above. From these fits, the standard error of the PSE and its 95% confidence interval were determined for each participant and each stimulus.

## 3 Results

### 3.1 Experiment 1

Example psychometric functions for one observer (SH) with a reference contrast value of 0.6 for each different probe are shown in Fig. 2. In these functions, the plaid was adapted in the right visual hemi-field and the components in the left. The *y*-axis reports the proportion of trials in which the subject reported the test probe to have higher apparent contrast than the reference probe. This is 0.5 when the stimuli appear equal (the PSE). The *x*-axis represents the percentage Michelson contrast required to equate the test and reference probe. In this instance, a shift in the PSE to the left represents additional contrast in the component-adapted field to make the probes look equal while a shift to the right indicates that additional contrast is required on the compound-adapted side. For the component probes, the PSE occurs when the probes are veridically similar, consistent with the roughly equal component adaptation in the two fields. For the plaid probe, however, the PSE occurs when the right-hand probe has a contrast of 0.76 versus 0.60 in the left, indicating that the plaid was more strongly adapted by the plaid pattern adaptor.

**Fig. 3:**
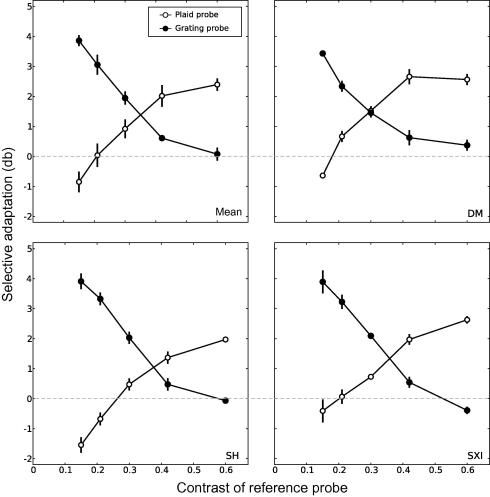
The additional contrast required for a test probe to appear equal to a reference probe after adaptation is plotted for both grating and plaid stimuli. Each data point represents the PSE, determined from a Weibull function fitted to data from 400 trials (200 on each side of fixation). For all subjects adaptation to the plaid is most apparent at high contrasts, while adaptation to the grating stimuli has greatest effect at low contrasts. For the mean data (upper left panel) the error bars represents ±1sem between subjects. For individual subjects the error bars represent ±1sem of the estimate of the PSE, calculated from 5000 bootstrap resamples of the data (see text for details). Dotted line denotes equal adaptation in component- and compound-adapted fields.

The overall adaptation effects for both component and compound adaptation are shown in Fig. 3 and are plotted as differential effects. Zero on these graphs indicates equal adaptation in both the component- and compound-adapted fields (i.e. no additional contrast is required in either field to equate the two probes), positive values indicate pattern-specific adaptation (for grating probes this is the additional contrast required in the component-adapted field to make it appear equal to one presented in the compound-adapted field, and vice versa for a plaid probe). Negative values indicate that a given probe was adapted more by the opposite stimulus, for instance, where adaptation was more apparent in the component-adapted field than the compound-adapted field for a plaid probe. Adaptation effects are plotted in decibels using the following equation:

$$\text{dB}=20*\log10(C_{\text{adapt}}/C)$$

where *C* is the contrast of the reference probe and *C*_adapt_ is the Michelson contrast value required to equate the test probe to the reference.

Data are shown for each individual participant and the average across all participants (top left panel). Consistent with previous studies, selective adaptation to the component gratings was most visible at low contrasts when plotted on logarithmic axes (Georgeson, 1985; Snowden & Hammett, 1992). It appears that the procedure of keeping identical time-averaged presentation of the component intensities in the two fields did not actually result in identical adaptation of the components, at least for certain (low) contrasts. Conversely, selective adaptation to the plaid was most apparent at high contrasts and this effect diminished as the contrast of the plaid probe decreased, until a contrast value of 0.15 where adaptation to the plaid was roughly equal in the two fields. Thus, high contrast plaid probes were more affected by adaptation to plaids than to gratings, whereas low contrast grating probes were more affected by adaptation to grating adaptors than plaids.

The paradigm used for this experiment acted to equate the Fourier energy in each adapted field. However, another procedure for keeping the stimulus exposure time equal in both fields is to equate the pixel contrast in each hemi-field. That is, adapt to alternating, full contrast grating stimuli in the component-adapted field, while adapting to a constant, full-contrast plaid in the compound field. To ensure that both procedures produce comparable results, all three observers were also tested for one reference contrast (0.3) with pixel contrast equated in both adapted fields. The results were very similar for each timing procedure; equating the Fourier energy of the stimuli led to a 1.3996 dB adaptation effect, while equating the pixel contrast led to 1.5836 dB effect. The similarity in the magnitude of adaptation suggests that both timing procedures produce comparable results.

### 3.2 Experiment 2

#### 3.2.1 Experiment 2A: phase tuning of grating probes

**Fig. 4:**
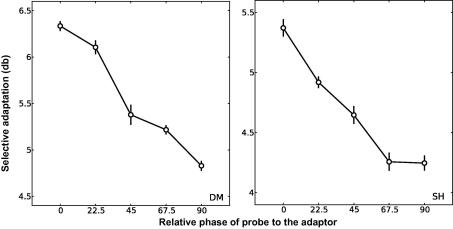
Contrast adaptation of a grating stimulus reduces as the phase of the probe moves further away from the adaptor indicating that the underlying mechanism is tuned for phase. Each data point represents the PSE, determined from a Weibull function fitted to data from 400 trials (200 on each side of fixation). Error bars represent ±1sem of the estimate of the PSE, calculated from 5000 bootstrap resamples of the data (see text for details).

To facilitate comparison of phase selectivity using grating and plaid stimuli, we first examined whether varying the spatial phase of the probe relative to adaptor had any influence on the effect of adaptation to a grating stimulus. The procedure in this experiment differed slightly from the other experiments. Participants adapted to a full contrast, counterphase modulated grating stimulus in one hemi-field, with no adaptor on the opposite side. Following adaptation participants compared the contrast of a grating probe in the adaptation location with one in the other visual hemi-field. Plaid stimuli were not tested in this experiment. Observers were tested with a reference probe of a fixed contrast (0.42). Reference and test probes took one of five phase values, ranging from 0° (identical to the adaptor) to 90° away from the adaptor. As in the other experiments the adaptor side was counterbalanced across runs so that 200 trials were collected for each condition on each side. Data are shown for two participants in Fig. 4. The *x*-axis reports the relative spatial phase of the probe to the adaptor, while the *y*-axis reports the adaptation effect in decibels as in Experiment 1. For both participants adaptation decreases markedly as the phase of the probe is moved away from the adaptor suggesting the underlying mechanism is tightly tuned for phase.

#### 3.2.2 Experiment 2B: phase tuning of plaid probes

**Fig. 5:**
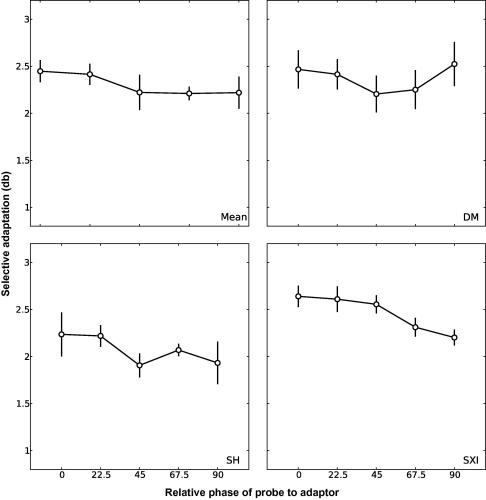
The relative spatial phase of the probe to the adaptor had no impact on the compound adaptation effect. Each data point represents the PSE, determined from a Weibull function fitted to data from 400 trials (200 on each side of fixation). For the mean data (upper left panel) the error bars represents ±1sem between subjects. For individual subjects the error bars represent ±1sem of the estimate of the PSE, calculated from 5000 bootstrap resamples of the data (see text for details).

In Experiment 2B we examined whether the relative spatial phase of the probe to the adaptor had any impact on adaptation to plaid patterns. The phase and contrast values of the reference and test probes were identical to those used in Experiment 2A. The adaptation procedure was similar to Experiment 1, whereby observers were adapted to a plaid in one visual hemi-field while simultaneously adapting to its components in the other. As in Experiment 1, observers were probed with both grating and plaid stimuli. However, both grating and plaid stimuli were counterphase modulated, as in Experiment 2A, rather than phase-jittered. Fig. 5 shows the adaptation effects for each individual participant and the average across all participants (top left panel). As in Fig. 4, adaptation is plotted as a function of the relative phase of the probe to the adaptor. For all observers, adaptation to the component gratings (data not shown) remains close to zero across all probe phases, consistent with roughly equal adaptation to the components in each visual hemi-field. There is, however, a strong adaptation effect towards the plaid pattern in the plaid-adapted field. In this experiment, however, the spatial phase of the probe had no impact on this adaptation effect, suggesting that a plaid mechanism is phase invariant.

## 4 Discussion

We have shown, using a contrast adaptation paradigm, the effects of stimulus contrast and spatial phase on the responses of plaid-form-selective mechanisms in the visual system. The method was based on that of Peirce and Taylor (2006) but is simpler and generates superior adaptation effects (their method found an average effect of around 3% contrast, whereas the effects described here are over 15% contrast in many conditions). Our results show that the selective adaptation to plaid stimuli is strongest with high-contrast probes while selective adaptation to grating stimuli is most apparent at low contrasts. Selective plaid adaptation also appears to be invariant to the spatial phase of the probe relative to the adaptor, unlike contrast adaptation seen for grating stimuli, which demonstrated a tight tuning for phase. Together these results suggest that the visual system is equipped with separate mechanisms for detecting grating and plaid patterns.

Plaid stimuli have been used as an investigative tool extensively in research on visual motion processing (Adelson & Movshon, 1982; Huk & Heeger, 2002; Movshon et al., 1986); while other studies have used plaids to study early interactions between visual channels (Carandini, Barlow, O’Keefe, Poirson, & Movshon, 1997; Georgeson & Meese, 1997). However, very little research has investigated the mechanisms that respond selectively to the *form* of the plaid pattern, as studied here. Such mechanisms are potential precursors to the plaid-motion-selective cells found in area MT/V5 (Movshon et al., 1986), although we suspect they are unrelated. Plaid motion might be detected by identifying the presence of particular combinations of gratings (plaids) and then determining their change in spatial position in time (i.e. their motion). Alternatively, the motion of each component might be detected independently and overall motion of the plaid determined from a combination of these motion signals (e.g. Welch, 1989). Recent work (Majaj, Carandini, & Movshon, 2007; Rust et al., 2006) has supported the latter hypothesis. Furthermore, the former model, in which the plaid form is detected prior to its motion, would be better served by phase-sensitive plaid detectors, whereas we find here a lack of dependence on spatial phase.

Since the constituent gratings were the same in the component- and compound-adapted fields (albeit with stimuli of different physical contrasts) we were expecting no difference in the apparent contrast of the grating probes in the two locations. In fact this was not the case. Component probes exhibited a positive shift in PSE, indicating that they showed more adaptation in the component-adapted field. Equally surprising was our finding that the magnitude of this adaptation was determined by the contrast of the test probes. The effect of the greater adaptation to the components on the component-adapted side was more apparent when the contrast of the probes was lower, whereas the plaid-selective adaptation was more apparent for high-contrast probes. For intermediate probes both stimuli were more adapted in their own respective field. These results cannot be explained by cross-orientation suppression (Morrone, Burr, & Maffei, 1982), as this process should lead to the plaid being the less salient adaptor leading to a smaller plaid adaptation effect, where critically we find greater adaptation to it at high contrasts. These results may, however, be partially explained by the finding that for grating stimuli the overall magnitude of adaptation is determined by the ratio of the adapting contrast to the test contrast (Georgeson, 1985). Thus, in this instance at low test contrasts (0.15, 0.21), a grating adaptor of 0.5 Michelson contrast leads to a strong adaptation effect. With high-contrast probes, however, the adapting/test ratio becomes smaller and a weaker adaptive effect is observed.

**Fig. 6:**
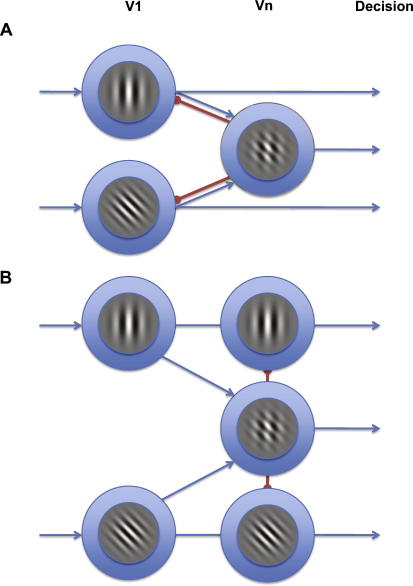
Two possible mechanisms to explain the inverse relationship between contrast adaptation to grating and plaid stimuli as a function of reference probe contrast. (A) At low test contrasts, adaptation to component stimuli is most apparent, consistent with other psychophysical findings. As the contrast of the test probe increases it approaches the optimal range for a plaid detector, which acts to inhibit the activity of the neurons responding to the grating stimulus in V1. (B) Alternatively, the mechanism mediating plaid-selective adaptation could inhibit the responses of the neurons responding to the component gratings through lateral inhibition at the level in which the plaid itself is encoded.

The above explanation does not, however, explain the changes in plaid-selective adaptation using different test contrasts; if it did we should again observe the greatest magnitude of plaid adaptation for low contrast plaid probes. Moreover, experiments from our laboratory that share a similar paradigm, but use adaptors with different contrasts, do not adhere to this ratio rule (data not shown). An alternative explanation for our data is that the visual system is equipped with separate neural mechanisms responsible for processing grating and plaid patterns that each have different contrast response properties. Indeed, previous research has suggested that contrast coding of these stimuli is different; when viewing a plaid and grating of an identical Michelson contrast an observer will perceive the plaid as lower in contrast (Cannon & Fullenkamp, 1991; Georgeson & Shackleton, 1994). Furthermore, the inverse relationship of adaptation observed for each stimulus type suggests an inhibitory influence between the two underlying mechanisms, where the response of each mechanism is modulated by the contrast of the test probe. That is, at high contrasts (optimal for plaid adaptation) a plaid-selective mechanism acts to inhibit the response of the mechanisms responsible for grating detection. This process could operate through feedback connections (e.g. Fang et al., 2008; Murray et al., 2002; Roach, Webb, and McGraw, 2008) whereby plaid-selective mechanisms inhibit the response of grating detectors once the contrast of the test probe reaches the optimal range for plaid detection (see Fig. 6A). Alternatively, this operation could occur through lateral inhibition, whereby the plaid-selective mechanism attenuates the response of the orientation-selective component detectors at the processing stage in which the plaid itself is encoded (see Fig. 6B).

**Fig. 7:**
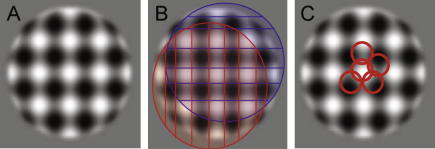
Two possible mechanisms for detecting a plaid pattern. Detection of the plaid (A) could be achieved through a mechanism, such as a logical AND gate, that combines the outputs from a small number of neurons with overlapping receptive fields covering a large area of the plaid pattern (B). Alternatively, sub-units with much smaller, neighbouring, receptive fields could respond to luminance patches within the plaid stimulus (C).

Although there is currently no physiological evidence for static plaid detectors, many studies have reported the presence of “pattern type” cells in area MT that respond to the overall motion of the plaid stimulus rather than responding to the motion of the individual component gratings (Born & Bradley, 2005; Movshon & Newsome, 1996; Movshon et al., 1986). It also seems likely that the mechanisms responsible for encoding static plaid patterns are located higher up the ventral stream than the grating or edge detectors of V1 (Olzak & Thomas, 1991, 1999). Visual areas V2 or V4 are well-placed to combine the outputs from V1, and are known to be sensitive to moderately complex patterns (Gallant et al., 1996; Ito & Komatsu, 2004; Pasupathy & Connor, 1999). The selective detection of plaids may result from the equivalent of a logical AND operation that results from the non-linear summation (Peirce, 2007a) or multiplication (Gheorghiu & Kingdom, 2009) of V1 outputs. Alternatively, it may be that V1 neurons with short, wide receptive fields are able to detect the form of the plaid pattern by responding to luminance patches within the stimulus (Derrington & Badcock, 1992; Tinsley et al., 2003) (see Fig. 7C). These possibilities may be distinguished by measuring the phase tuning of the adaptation effects. If plaids were detected by their local luminance cues then a shift in the relative spatial phase of the probe would act to reposition the location of these cues, resulting in other (non-adapted) mechanisms responding to the probe. Thus, the selective adaptation to the plaid should diminish as the spatial phase of the probe shifts further from that of the adaptor. We find this not to be the case. The selective adaptation to the plaid stimulus was unaffected by the spatial phase of the probe stimulus. This is more consistent with a plaid detector that combines the outputs of two V1 cells, which are likely to be complex, given the phase insensitivity observed here, that cover a large area of the stimulus with their receptive fields (see Fig. 7B).

In summary, we have shown that the selective adaptation of subjects to plaid patterns is qualitatively different from that to grating stimuli. The selective adaptation to gratings is greater for low-contrast probes, whereas for plaid patterns high-contrast probes demonstrate the greater selectivity. The mechanisms underlying the selective adaptation to plaids appear to be entirely insensitive to the spatial phase of the probe relative to its adaptor. We conclude that they are constructed from the non-linear combination of V1 complex cell outputs. There is a great deal still to know about these mechanisms. In particular we need to know where they reside anatomically, and how they relate to perceptual processes such as binding and figure-ground segregation.

## Appendix A Supplementary material

Supplementary Movie 1 Demonstration of the selective adaptation to plaids. While fixating the central point, the left adaptation field is adapted to alternating gratings, while the right is adapted to equivalent components presented together as a plaid. After adaptation, an identical plaid probe presented in the two locations appears lower contrast in the compound-adapted field.
